# Association of repeated high serum osmolarity with cognitive function in older Japanese adults in a KOBE study subanalysis

**DOI:** 10.1038/s41598-025-20607-4

**Published:** 2025-10-21

**Authors:** Tomofumi Nishikawa, Naomi Miyamatsu, Aya Higashiyama, Yoshimi Kubota, Yoko Nishida, Takumi Hirata, Aya Hirata, Junji Miyazaki, Yukako Tatsumi, Daisuke Sugiyama, Kazuyo Kuwabara, Sachimi Kubo, Yoshihiro Miyamoto, Tomonori Okamura

**Affiliations:** 1 Faculty of Health Science, Kyoto Koka Women’s University, 38 Kadonocho, Nishikyogoku, Ukyo-ku, 615-0822 Kyoto, Japan; 2 Department of Clinical Nursing, Shiga University of Medical Science, Shiga, Japan; 3 Department of Hygiene, Wakayama Medical University, Wakayama, Japan; 4 School of Medicine, Department of Preventive Medicine, Hyogo Medical University, Hyogo, Japan; 5 Osaka Institute of Public Health, Osaka, Japan; 6 Human Care Research team, Tokyo Metropolitan Institute for Geriatrics and Gerontology, Tokyo, Japan; 7 Department of Preventive Medicine and Public Health, School of Medicine, Keio University, Tokyo, Japan; 8 Public Health, Department of Social Medicine, Graduate School of Medicine, University of Osaka, Suita, Osaka Japan; 9 Department of Hygiene and Public Health, School of Medicine, Teikyo University, Tokyo, Japan; 10 Faculty of Nursing And Medical Care, Keio University, Tokyo, Japan; 11 Department of Public Health, Institute of Science Tokyo, Tokyo, Japan; 12 Department of Public Health, Kindai University Faculty of Medicine, Osaka, Japan; 13 Open Innovation Center, National Cerebral and Cardiovascular Center, Osaka, Japan; 14 Foundation for Biomedical Research and Innovation, Hyogo, Japan

**Keywords:** Dehydration, Serum osmolarity, Cognitive decline, Retrospective cohort study, Neurological disorders, Public health

## Abstract

The relationship between serum osmolarity and cognitive function has not been fully characterized. This study aimed to examine the cross-sectional association between repeated high serum osmolarity and cognitive performance among elderly community residents. We performed a subanalysis of the Kobe Orthopedic and Biomedical Epidemiological Study, including residents aged ≥ 75 years who completed the Japanese Montreal Cognitive Assessment (MoCA-J) in 2016–2017 (*n* = 127), 2018–2019 (*n* = 71), and 2020 (*n* = 16). Serum osmolarity was obtained from the data in the 2012–2013 survey and in the 2016–2017 survey. MoCA-J scores were dichotomized at ≤ 22 versus > 22. Multivariate logistic regression adjusted for demographic, lifestyle including daily non-alcohol drink intake, seasonal, and clinical covariates to assess associations between osmolarity status and cognitive group. Among 214 participants (mean age 76.2 ± 1.3 years; 56% female), high osmolarity (≥ 300 mOsm/L) in 2012–2013 was associated with MoCA-J ≤ 22 (OR 2.67, 95% CI 1.29–5.53, *p* = 0.008). A similar association emerged for 2016–2017 measurements (OR 6.12, 95% CI 1.46–25.61, *p* = 0.013). Participants with high serum osmolarity at both time points showed a stronger cross-sectional association with lower MoCA-J scores (OR 17.64, 95% CI = 1.8–184.83, *p* = 0.017). No significant association was observed between daily non-alcoholic drink (NAD) intake and either MoCA-J scores or serum osmotic pressure. Repeated high serum osmolarity was cross-sectionally associated with lower cognitive performance in Japanese community-dwelling older adults. While NAD intake showed no significant association, further research is needed to explore the potential role of serum osmolarity in cognitive health. These findings warrant confirmation in larger prospective studies.

## Introduction

The aging population is at increased risk for cognitive decline and related conditions such as dementia^1^. Identifying modifiable risk factors is crucial for developing preventive strategies. Previous studies have identified several modifiable and non-modifiable contributors, including age, sex, educational level, physical inactivity, smoking, alcohol consumption, hypertension, diabetes mellitus, hyperlipidemia, depression, and genetic predisposition such as the presence of the APOE ε4 allele^1,2^. However, information regarding body fluid balance and the risk of future cognitive impairment and dementia is insufficient^3^. Additionally, the impacts of global warming, such as rising temperatures and extreme weather events, may further complicate the health and well-being of older adults, potentially exacerbating risks related to hydration and cognitive function^4–6^.

Water-loss dehydration can be caused by inadequate fluid intake, excessive.

sweating or transcutaneous evaporation, and/or vomiting^7^. Serum osmolarity is a valuable indicator for assessing hydration status and is used as a definitive diagnosis (reference standard) for water-loss dehydration, as serum and intracellular osmolarity are so central to body fluid control that they act as a trigger to both thirst and renal conservation of fluid^8^. In older adults, maintaining body fluid volume becomes challenging due to difficulties in balancing water and sodium^9^.

Several studies indicate that poor hydration status is associated with cognitive dysfunction^10–17^, while it is still controversial^18^. However, while many studies have focused on cross-sectional research or transient effects, little is known about effects of the long-term hydration status on cognitive performance in healthy community-dwelling older adults. Moreover, the causal relationship between serum osmolarity and cognitive function remains unclear.

Japan is one of the world’s most rapidly aging societies, with adults aged 75 and older comprising over 14% of its population^19^. It also experiences some of the highest average summer temperatures and humidity levels globally, conditions that elevate dehydration risk. These demographic and climatic characteristics make Japan a relevant setting to explore the association between serum osmolarity and cognitive function in older adults. Therefore, this study aimed to examine whether repeated high serum osmolarity, observed across two time points, was cross-sectionally associated with cognitive performance in a healthy population aged 75 years and older.

## Methods

### Study design and setting

**Fig. 1:**
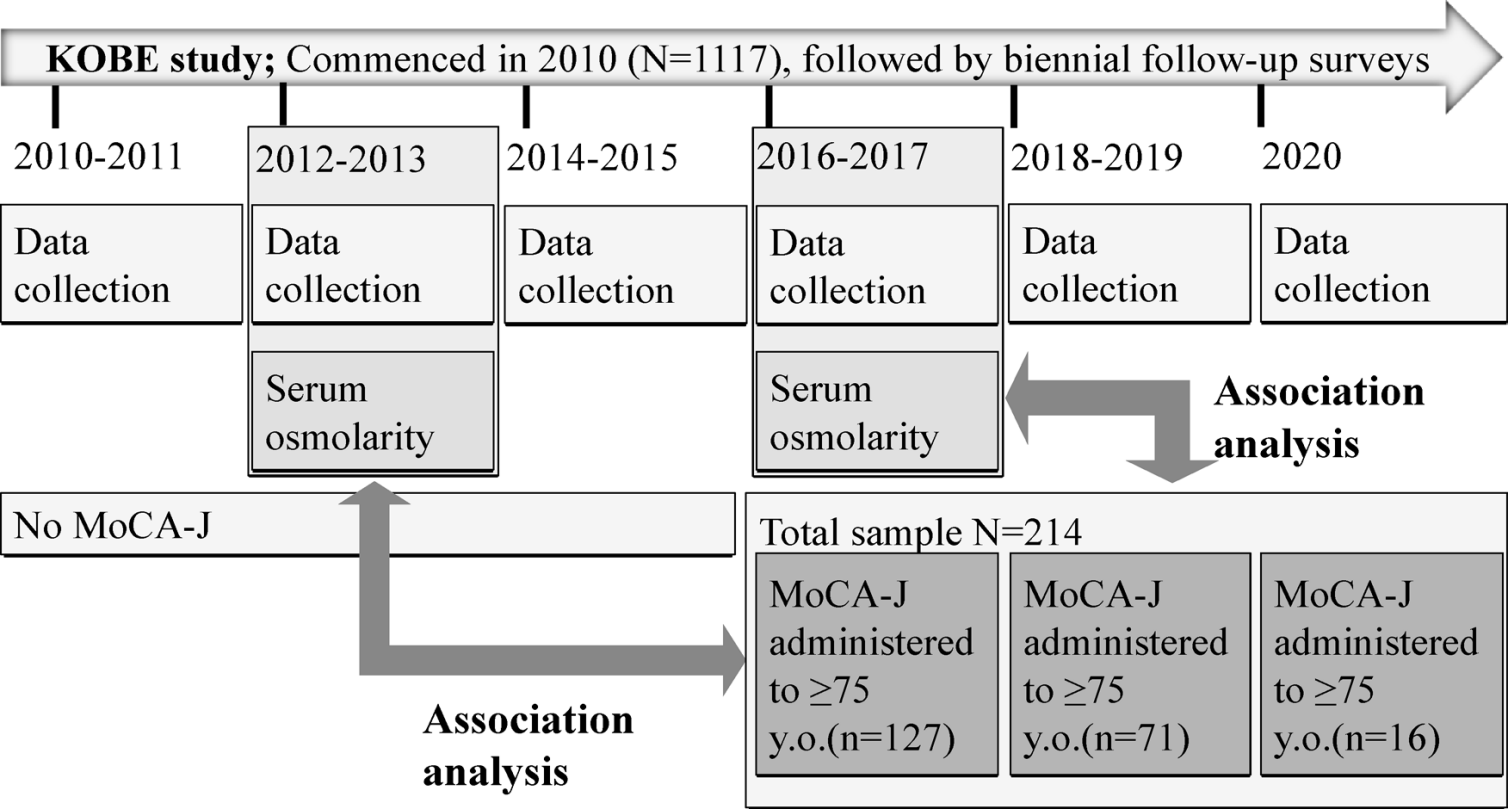
Participant selection and study design. This figure illustrates the timeline and data availability of the Kobe Orthopedic and Biomedical Epidemiologic (KOBE) Study, a longitudinal cohort study initiated in 2010 with biennial follow-up surveys. Serum osmolarity was measured during the 2012–2013 and 2016–2017 surveys. A subgroup of 214 participants aged ≥ 75 years who underwent the Japanese version of the Montreal Cognitive Assessment (MoCA-J) in one of three survey periods (2012–2013, 2016–2017, or 2020) was selected for analysis. Thick arrows indicate the periods and participant groups used to examine the relationship between MoCA-J scores and serum osmolarity.

This study is a subgroup analysis using data from participants in the Kobe Orthopedic and Biomedical Epidemiological (KOBE) study aged ≥ 75 years who underwent the Japanese version of the Montreal Cognitive Assessment (MoCA-J) testing in the 2016–2017 (*n* = 127), 2018–2019 (*n* = 71), and 2020 (*n* = 16) surveys (Fig. 1). In this study, we conducted two analyses using MoCA-J scores obtained during the 2016–2020 surveys: (1) an analysis linking serum osmolarity measured in the 2012–2013 survey to these MoCA-J scores; (2) an analysis linking serum osmolarity measured in the 2016–2017 survey to these MoCA-J scores; and (3) an analysis examining the association between the number of times a participant exceeded the high-osmolarity threshold across both surveys (none, either one, or both) and their MoCA-J score.

We included both time points in the analysis to account for potential cumulative effects of serum osmolarity on cognitive function. This approach enables us to examine the possible impact of chronic exposure, given that persistently elevated osmolarity across surveys may reflect long-term physiological stress. Although baseline cognitive data were unavailable in earlier surveys, this design allows for exploratory assessment of temporal patterns.

### Study population

The KOBE study is a population-based prospective cohort study of risk factors for cardiovascular disease or worsening of quality of life in Kobe, a major urban area located in west Japan^20,21^. The KOBE study commenced in 2010, and during the baseline survey conducted from July 2010 to December 2011, 1,117 subjects (776 women and 341 men) participated. The study participants were volunteers aged 40 to 74 years who were residents of Kobe; the participants had to meet the following criteria: (1) not currently on medications for hypertension, dyslipidemia, and diabetes mellitus; (2) no history of cardiovascular diseases and cancer; (3) perceive themselves as subjectively healthy; (4) be able to independently travel to the examination site; and (5) voluntarily agree to participate in the study as a research volunteer. Follow-up surveys were conducted biennially after the baseline survey, as previously reported^21–25^. In the present sub-analysis, we included participants who met the baseline criteria but subsequently developed hypertension, dyslipidemia, or diabetes mellitus and began medication during follow-up. This reflects the real-world health transitions captured in the longitudinal design of the KOBE Study. This sub-analysis focused on participants who underwent MoCA-J assessments between 2016 and 2020 and were aged ≥ 75 years at the time of the assessment. These individuals were retrospectively selected from the original KOBE study based on the availability of cognitive and serum osmotic pressure data. MoCA-J was not implemented until the 2016–2017 survey; hence, no objective dementia or MCI data exist for the baseline or 2012–2013 follow-ups. In Kobe, there is a clear difference in temperature depending on the season. For instance, the monthly mean temperatures in 2012 (annual average temperature 16.6℃) are as follows: in January 5.7℃, in February 5.2℃, in March 9.1℃, in April 15.2℃, in May 19.4℃, in June 23.0℃, in July 27.2℃, in August 29.3℃, in September 26.2℃, in October 19.8℃, in November 12.7℃, and in December 6.8℃^26^. To account for seasonal effects on hydration status and cognitive performance, survey season was categorized into “warmer” (May–October) and “colder” (November–April) periods, based on our previous study [35], and included as a categorical variable in the analyses.

### Data collection for characteristics

The data collection procedures were consistent with those detailed in our prior studies^21–24^. Data collection was conducted every month except for April and August in the 2012–2013 survey, and every month except for April in the 2016–2017 survey. Each participant was divided into one of these months, and completed a self-reported questionnaire to assess past medical history and life habits, including current smoker, current drinker, and daily non-alcohol drink (NAD) intake. Evaluation of daily NAD intake was detailed in our prior studies^27^. The questionnaire covered coffee, green tea, black tea, barley tea, water, milk and milk-based beverages, isotonic drinks, soy milk, and other non-alcoholic beverages. Total NAD intake was calculated as the average daily volume (mL/day) of these beverages, explicitly excluding all alcoholic drinks. Height and body weight were measured with patients wearing socks and light clothing. The blood pressure was measured twice in each participant after a five-minute rest using an automatic sphygmomanometer (Nihon Colin, BP-103iⅡ), and the mean value for each participant was recorded. All blood samples were obtained in the morning after fasting for at least 10 h, and blood and urine samples were tested by one commissioned clinical laboratory center (SRL Inc., Tokyo, Japan). MoCA-J was implemented only in 2016–2020 surveys.

### Exposure measurement: serum osmolarity/osmolality

In the 2012–2013 survey, serum osmolarity (mOsm/L) was calculated using Worthley’s formula: 2 × (serum sodium in mEq/L) + (BUN in mg/dL)/2.8 + (glucose in mg/dL)/18^28^. On the other hand, during the 2016–2017 survey, serum osmolality (mOsm/kg) was directly measured using freezing point osmometry (SRL Inc., Tokyo, Japan); however, calculated serum osmolarity could not be determined, as measurements for serum sodium and BUN were not performed. Usually, calculated serum “osmolarity” is an estimation of the osmolar concentration of serum and is proportional to the number of particles per liter of solution; it is expressed as mOsm/L, while directly measured serum “osmolality” is defined as the osmotic concentration of blood serum, expressed as the number of milliosmoles of solute per kilogram of serum water (mOsm/kg)^29^.Plasma density varies between 1.020 and 1.030 kg/L, yielding an average conversion factor of 1.026 (osmolality × 1.026 ≈ osmolarity) with < 3% difference in practice^30^. Taking this into account, we analyzed measured serum osmolality (mOsm/kg) and calculated serum osmolarity (mOsm/L) separately; however, for notational simplicity, unless there is a specific need to differentiate, osmolarity will be used to refer to osmotic pressure in the present study.

### Statistical analysis

The correlation between the MoCA-J scores and serum osmolarity, as measured in the 2012–2013 and 2016–2017 surveys, was analyzed. We used Pearson’s correlation coefficient to assess the correlation between equivalent test results obtained in each survey. Subsequently, we investigated the association between the MoCA-J scores and the results of the serum osmotic pressure obtained in the 2012–2013 and the 2016–2017 surveys, using logistic regression models. In these models, MoCA-J scores were dichotomized into ≤ 22 and > 22^31,32^. In this study, thresholds for relatively high serum osmolarity were set at 300 mOsm/L or 295 mOsm/kg according to past research^29,33^, and the conversion factor^30^. The analyses in model 1 adjusted for age at the time of the MoCA-J assessment and sex, and the analyses in model 2 adjusted for the following variables: age at the time of the MoCA-J assessment, sex, height, weight, survey season (categorized as warmer [May–October] vs. colder [November–April]), current smoker, current drinker, systolic blood pressure (SBP), diastolic blood pressure (DBP), hypertension (systolic blood pressure ≥ 140 mmHg, diastolic blood pressure ≥ 90 mmHg, or taking antihypertensive medication), dyslipidemia (LDL ≥ 140 mg/dL, triglycerides ≥ 150 mg/dL, HDL < 40 mg/dL or taking medication for dyslipidemia)^34^, diabetes (fasting glucose ≥ 126 mg/dL, HbA1c ≥ 6.5% or taking medication for diabetes), and NAD intake. These variables were defined based on clinical thresholds and current medication use at the time of each survey, regardless of baseline status. This approach reflects the longitudinal nature of the cohort and allows for inclusion of participants who began treatment during follow-up. In this sub-analysis, we included only participants who underwent MoCA-J assessments between 2016 and 2020. For these individuals, serum osmolarity data from the 2012–2013 and 2016–2017 surveys were linked. This approach allowed us to incorporate serum osmolarity data from multiple time points, enabling a more comprehensive evaluation of its association with cognitive function at the time of MoCA-J assessment. Although the broader cohort was longitudinal in design, this sub-analysis was cross-sectional in nature, focusing on associations between serum osmolarity and cognitive performance measured at a single time point. Given that the age range of participants was relatively narrow (75–80 years, as shown in Table 1), age was treated as a continuous variable in the logistic regression models. We did not set a specific cutoff for age, as categorizing a narrow age range could lead to unnecessary information loss and reduced statistical efficiency. Furthermore, we examined similar logistic regression models by categorizing individuals into three groups: those not considered to have high serum osmotic pressure in either survey, those who exceeded the threshold for high serum osmotic pressure in one survey, and those who exceeded the threshold in both surveys.

**Table 1:** The data from each survey.

		Women						Men						Total					
		S.D.			Range			S.D.			Range			S.D.			Range		
The subjects in this subgroup analysis	Number	120 (56.1%)						94 (43.9%)						214					
	MoCA-J (2016–2020)	24.8 ± 3.2			14.0 - 30.0			24.2 ± 3.3			13.0 - 30.0			24.5 ± 3.2			13.0 - 30.0		
	Age at MoCA-J	76.1 ± 1.2			75 - 80			76.3 ± 1.3			75 - 80			76.2 ± 1.3			75 - 80		
The 2012–2013 survey	Number	120 (56.1%)						94 (43.9%)						214					
	Age	71.2 ± 2.1			67 - 76			71.5 ± 2.0			68 - 76			71.3 ± 2.1			67 - 76		
	Height	153.2 ± 5.0			140.9 - 166.4			164.6 ± 5.9			146.2 - 177.1			158.2 ± 7.8			140.9 - 177.1		
	Weight	50.3 ± 7.5			35.9 - 80.1			61.1 ± 8.4			43.2 - 83.2			55.1 ± 9.5			35.9 - 83.2		
	Smoker at the survey	0 (0.0%)						6 (6.4%)						6 (2.8%)					
	Drinker at the survey	30 (25.0%)						76 (80.9%)						106 (49.5%)					
	SBP (mmHg)	118.2 ± 17.2			75.0 - 161.0			122.0 ± 17.4			90.0 - 174.0			119.9 ± 17.4			75.0 - 174.0		
	DBP (mmHg)	69.9 ± 10.3			45.0 - 97.0			75.2 ± 10.0			56.0 - 106.0			72.2 ± 10.5			45.0 - 106.0		
	Hypertension	22 (18.3%)						27 (28.7%)						49 (22.9%)					
	Diabetes	2 (1.7%)						6 (6.4%)						8 (3.7%)					
	Dyslipidemia	65 (54.2%)						31 (33.0%)						96 (44.9%)					
	NAD intake (mL/day)	1837.8 ± 795.4			450.0 - 4680.0			1684.3 ± 560.6			400.0 - 3150.0			1770.3 ± 704.6			400.0 - 4680.0		
	Calculated serum osmolarity (mOsm/L)	298.2 ± 3.3			290.6 - 307.4			296.9 ± 3.6			287.8 - 304.8			297.6 ± 3.5			287.8 - 307.4		
The 2016–2017 survey	Number	113 (55.1%)						92(44.9%)						205					
	Age	75 ± 2			71 - 80			76 ± 2			72 - 80			75 ± 2			71 - 80		
	Height	152.2 ± 5.1			140.4 - 165.0			163.9 ± 6.0			146.4 - 176.7			157.4 ± 8.0			140.4 - 176.7		
	Weight	49.9 ± 7.4			36.2 - 76.7			61.1 ± 9.1			40.8 - 83.5			55.0 ± 9.9			36.2 - 83.5		
	Smoker at the survey	0 (0%)						6 (6.50%)						6 (2.90%)					
	Drinker at the survey	32 (28.3%)						71 (77.2%)						103 (50.2%)					
	SBP (mmHg)	120.9 ± 17.8			81.0 - 165.0			125.3 ± 18.8			83.0 - 171.0			122.9 ± 18.4			81.0 - 171.0		
	DBP (mmHg)	69.8 ± 10.3			47.0 - 96.0			73.4 ± 9.5			55.0 - 99.0			71.4 ± 10.1			47.0 - 99.0		
	Hypertension	14 (12.4%)						20 (21.7%)						34 (16.6%)					
	Diabetes	58 (51.3%)						41 (44.6%)						99 (48.3%)					
	Dyslipidemia	5 (4.4%)						7 (7.6%)						12 (5.9%)					
	NAD intake (mL/day)	1791.2 ± 691.0			0.0 - 4350.0			1575.0 ± 650.1			300.0 - 3400.0			1694.2 ± 679.9			0.0 - 4350.0		
	Measured serum osmolality (mOsm/kg)	287.9 ± 3.4			278 - 299			288.0 ± 4.0			272 - 297			287.9 ± 3.6			272 - 299		

Continuous data was analyzed using student’s t test and is shown in the mean ± standard deviation. S.D.; standard deviation. Categorical data was analyzed using the chi-square test and is shown as number (%). Hypertension; systolic blood pressure > = 140mmHg, diastolic blood pressure > = 90, or taking antihypertensives. Diabetes; fasting glucose > 126 mg/dL, HbA1C > = 6.5%, taking anti-diabetics medication or its history in the medical record. Dyslipidemia; LDL > = 140 mg/dL, triglyceride > = 150 mg/dL or HDL < 40 mg/dL. LDL; low density lipoprotein. HDL; high density lipoprotein. NAD; non-alcohol drink. MoCA-J; Japanese version of Montreal Cognitive Assessment, conducted between 2016 and 2020. No MoCA-J assessments were conducted during the 2012–2013 survey.

All significance tests were two-tailed, and *p* < 0.05 was considered significant in all analyses. The mean value was expressed as mean ± significant difference (S.D.). A paired t-test was used to detect differences between continuous variables across each survey. All statistical analyses were performed with IBM SPSS Statistics for Windows version 25 (IBM Corp., Armonk, NY).

### Ethics approval and consent to participate

This study was conducted according to the guidelines laid down in the Declaration of Helsinki, and all procedures involving research study participants were approved by the Ethics Committees of the Institute of Biomedical Research and Innovation (Committee approval number: 11–12) and Kyoto Koka Women’s University (Committee approval number: 012). Written informed consent was obtained from all patients.

## Results

Characteristics of the subjects stratified by sex are presented in Table 1. Additional demographic and clinical characteristics of the subjects, including comparisons across serum osmolarity groups and survey years, are provided in Supplementary Table S1. The average MoCA-J score was 24.5 ± 3.2 (24.8 ± 3.2 for women and 24.2 ± 3.3 for men). Among the participants, 120 individuals scored 23 or higher, while 94 individuals scored 22 or lower. The average age at the time of MoCA-J assessment was 76.2 ± 1.3 years (76.1 ± 1.2 for women and 76.3 ± 1.3 for men). The mean follow-up period from the 2012–2013 survey to the MoCA-J assessment was 1,800 days (approximately 4.9 years, range: 1078–2716 days). The month in which each individual’s survey was conducted was not intentionally assigned in any follow-up surveys, so there was no significant correlation in the surveyed month between the 2012–2013 survey and the 2016–2017 survey; the difference in survey months between the two surveys averaged 2.4 months (ranging from 0 to 6 months). There was no significant difference in daily NAD intake for each individual between the 2012–2013 survey and the 2016–2017 survey (paired t-test). Furthermore, a significant positive correlation was observed in individuals’ daily NAD intake between the two surveys (*r* = 0.540, *p* < 0.001), indicating consistency in their intake patterns, despite differences in the survey months. The mean serum osmolarity (calculated) was 297.6 ± 3.5 mOsm/L in the 2012–2013 survey, and it (measured) was 287.9 ± 3.6 mOsm/kg in the 2016–2017 survey, corresponding to an estimated osmolarity of 295.4 ± 3.7 mOsm/L using a plasma density factor of 1.026 kg/L^30^. There were no individuals reporting any symptoms of dehydration.

**Fig. 2:**
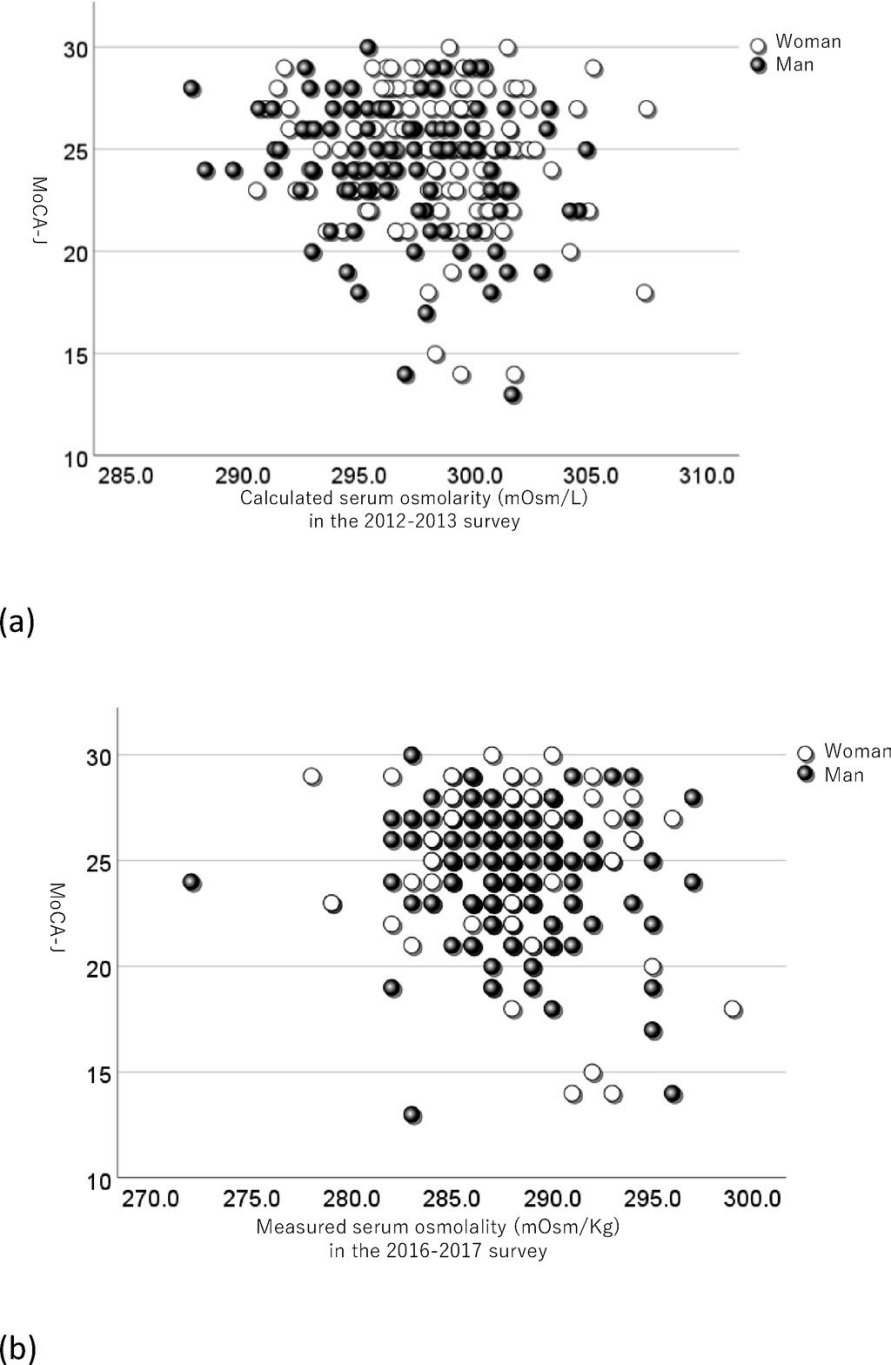
The scatter plots illustrating the relationship between serum osmotic pressure and MoCA-J among participants. Each point represents an individual, with serum osmolarity on the x-axis and MoCA-J on the y-axis. (**a**). The relationship between serum osmotic pressure obtained from the 2012–2013 survey and the MoCA-J score. (**b**). The relationship between serum osmotic pressure obtained from the 2016–2017 survey and MoCA-J. MoCA-J; Japanese version of Montreal Cognitive Assessment, which was assessed between 2016 and 2020. In the 2012–2013 survey, calculated serum osmolarity was obtained. In the 2016–2017 survey, measured serum osmolality was obtained.

**Fig. 3:**
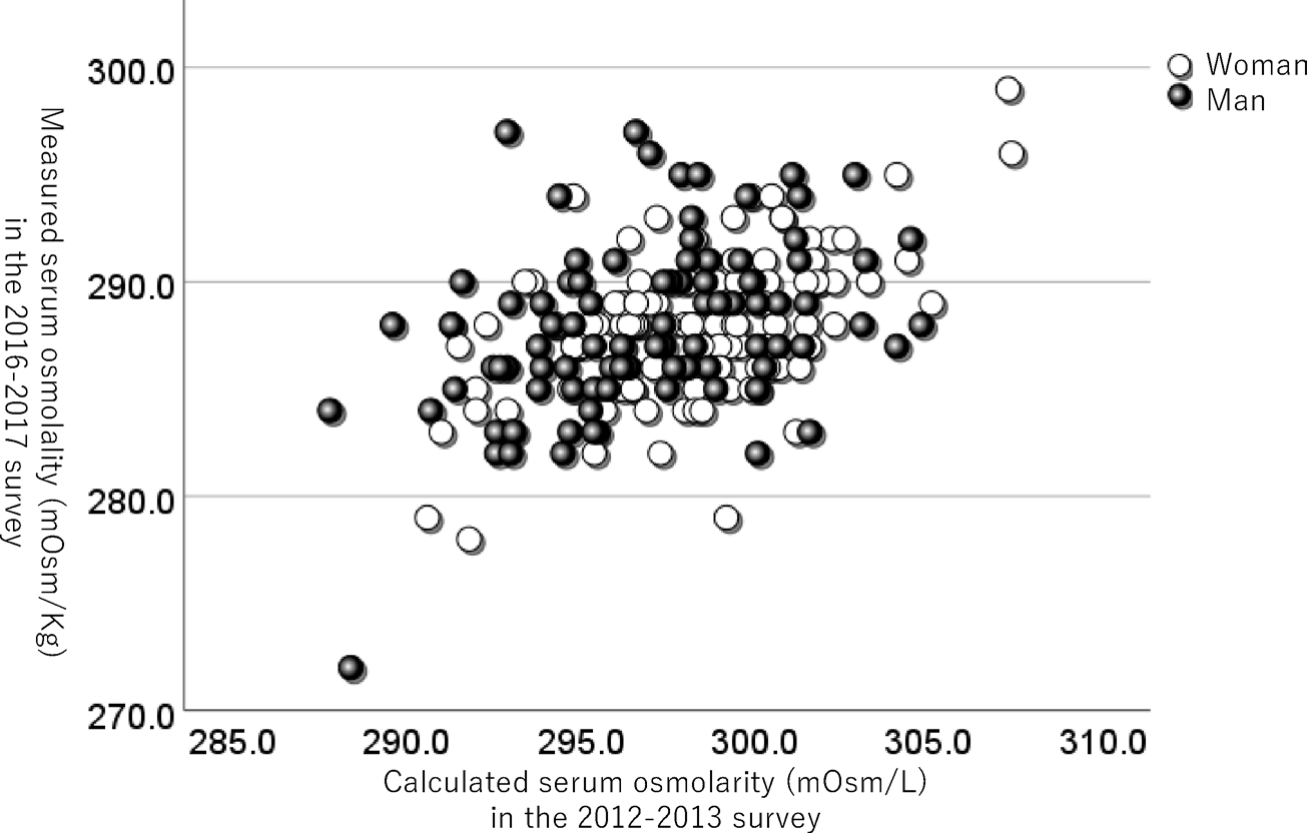
The scatter plot comparing serum osmotic pressure between participants from the 2012–2013 and 2016–2017 surveys. Each point represents an individual, with osmotic pressure from the 2012–2013 survey on the x-axis and osmotic pressure from the 2016–2017 survey on the y-axis. MoCA-J; Japanese version of Montreal Cognitive Assessment, which was assessed between 2016 and 2020. In the 2012–2013 survey, calculated serum osmolarity was obtained. In the 2016–2017 survey, measured serum osmolality was obtained.

When plotting the relationship between serum osmotic pressure and MoCA-J on a scatter plot in the 2012–2013 and the 2016–2017 surveys, it was observed that individuals with higher serum osmotic pressure tended to have lower MoCA-J scores (Fig. 2a and b). In addition, despite the differences in testing methods and survey months between the two surveys, a positive correlation was observed in serum osmotic pressure across both surveys (*r* = 0.440, *p* < 0.001, Fig. 3).

Both univariate and multivariate analyses revealed a statistically significant association between MoCA-J and serum osmotic pressure obtained in the 2012–2013 survey or those in the 2016–2017 survey (Table 2). In the multivariate logistic regression model 2, using the data in the 2012–2013 survey, the odds ratio (OR) was 2.67 (95% CI 1.29–5.53, *p* = 0.008). Even when the analysis was conducted using serum osmotic pressure from the 2016–2017 survey, similar results were obtained (OR 6.12, 95% CI 1.46–25.61, *p* = 0.013). Furthermore, individuals who exhibited high serum osmotic pressure in both the 2012–2013 and 2016–2017 surveys tended to have MoCA-J ≤ 22 (OR 17.64, 95% CI 1.68–184.83, *p* = 0.017). None of the additional covariates achieved statistical significance (all *p* > 0.05). To confirm the robustness of the adjusted model, sensitivity analyses excluding low-prevalence variables (e.g., smoking) were conducted, and similar results were obtained (e.g., OR = 2.679, 95% CI: 1.296–5.536, *p* = 0.008), supporting the stability of the primary findings.

**Table 2:** Multivariate logistic regression models of serum osmotic pressure for lower MoCA-J.

		Univariate			Model 1			Model 2		
Serum osmotic pressure	N(MoCA-J=<22)	OR	95% CI	p value	OR	95% CI	p value	OR	95% CI	p value
The 2012-2013 survey	N=214									
<300mOsm/L	28/154 (18.2%)	ref.			ref.			ref.		
>=300mOsm/L	20/60 (33.3%)	2.25	1.15-4.4	0.019	2.27	1.15-4.50	0.019	2.67	1.29-5.53	0.008
The 2016-2017 survey	N=205									
<295mOsm/Kg	38/195 (19.5%)	ref.			ref.			ref.		
>=295mOsm/Kg	6/10 (60.0%)	6.2	1.67-23.05	0.007	6.16	1.63-23.28	0.007	6.12	1.46-25.61	0.013
The number of times of high serum osmotic pressure observed across the two surveys										
None	26/145 (17.9%)	ref.			ref.			ref.		
Either	14/55 (25.5%)	1.56	0.71-3.51	0.237	1.57	0.74-3.30	0.239	1.57	0.71-3.51	0.268
Both	4/5 (80%)	18.31	1.67-170.60	0.011	18.6	1.99-173.82	0.01	17.64	1.68-184.83	0.017

The MoCA-J and age data were taken from the MoCA-J assessment conducted between 2016 and 2020. For other data, results from the 2012–2013 survey were used for analyses related to the 2012–2013 survey, and results from the 2016–2017 survey were used for analyses related to the 2016–2017 survey and for analyses related to the number of times within the high range of serum osmotic pressure in both surveys. Age and sex were adjusted in model 1. Age, sex, the survey season (warmer [May–October] vs. colder [November–April]), height, weight, non-alcohol drink intake, drinking history, smoking history, systolic blood pressure, diastolic blood pressure, hypertension, diabetes, and dyslipidemia were adjusted in model 2.95% CI: 95% confidence interval. MoCA-J: Japanese version of Montreal Cognitive Assessment. Hypertension: systolic blood pressure ≥140 mmHg, diastolic blood pressure ≥90 mmHg, or taking antihypertensives. Diabetes: fasting glucose 126 mg/dL, HbA1C ≥6.5%, taking anti-diabetic medication or its history in the medical record. Dyslipidemia: LDL ≥140 mg/dL, triglyceride ≥150 mg/dL, or HDL 40 mg/dL. LDL: low-density lipoprotein. HDL: high-density lipoprotein. Serum osmotic pressure: calculated serum osmolarity in the 2012–2013 survey and measured serum osmolality in the 2016–2017 survey. The number of times of high serum osmotic pressure observed across the two surveys was counted based on exceeding the threshold in each survey. OR: Odds ratio.

In this series of analyses, no significant association was observed between daily NAD intake and MoCA-J scores. Furthermore, daily NAD intake was also not significantly associated with serum osmotic pressure.

## Discussion

This study is the first to suggest that repeated observation of elevated serum osmotic pressure may be cross-sectionally associated with lower cognitive performance in a general elderly population. We found that serum osmolarity measured in general residents in their early 70s was associated with MoCA-J scores ≤ 22 assessed approximately five years later. Additionally, serum osmolarity measured closer to the time of cognitive assessment showed similar trends. Notably, individuals with elevated serum osmotic pressure at both the 2012–2013 and 2016–2017 surveys exhibited the strongest association with MoCA-J ≤ 22, suggesting that persistent elevation may be linked to cognitive status. In this study, repeated elevation was defined as serum osmotic pressure exceeding the threshold at two independent time points. Although MoCA-J assessments were not conducted prior to 2016, the consistency of associations across both time points indicates a pattern worth further investigation. While we acknowledge that the absence of baseline cognitive data limits causal interpretation, including both time points allowed us to explore temporal patterns and strengthen the overall evidence for a possible link between serum osmolarity and cognitive function. On the other hand, no significant associations were observed between daily NAD intake and either MoCA-J scores or serum osmotic pressure.

To date, there have been few studies investigating the relationship between serum osmotic pressure and cognitive function. A study on 28 healthy community-dwelling older adults demonstrated lower hydration status was related to slowed psychomotor processing speed and poorer attention/memory performance^13^. A recent cross-sectional study of 2,506 community-dwelling older adults (aged 60 and above) found that relatively low hydration status (with serum osmolarity between 285 and 289 mmol/L) was moderately associated with attention/processing speed among women but not among men by using multiple linear regression models^14^. The only prospective cohort study, observing 1,957 adults (aged 55–75) with overweight/obesity (BMI between ≥ 27 and < 40 kg/m^2^) and metabolic syndrome for 2 years, demonstrated that lower physiological hydration status (i.e., greater serum osmolarity) was associated with a greater decline in global cognitive function^15^. Building on these findings, our results suggest that repeated elevation of serum osmotic pressure may be associated with lower cognitive performance in healthy older adults. Although age-related decline in thirst regulation and drinking behavior may plausibly contribute to increased serum osmotic pressure, our previous study based on the entire KOBE study cohort demonstrated that age was independently associated with elevated serum osmolarity, whereas NAD intake did not vary significantly with age^35^. Combined with the findings of the present study, in which NAD intake was not significantly associated with either MoCA-J scores or osmotic pressure, these results suggest that behavioral factors such as drinking habits may only partially explain osmotic pressure changes in the elderly, and other physiological mechanisms may play a more substantial role. Adequate fluid intake is considered an effective strategy for preventing dehydration and regulating serum osmolarity. Although no significant association was observed in the present study, further research is warranted to clarify the role of hydration behaviors in cognitive health among older adults.

Dehydration and high osmolarity are believed to have a stronger impact on older individuals than on younger ones^36,37^. For example, a study, comparing a younger group (*N* = 9, age 24 ± 3 years) and an older group (*N* = 8, age 56 ± 3 year) after 10 consecutive days of high-intensity hill walking, demonstrated that the younger group remained hydrated, whereas the older group became progressively dehydrated, indicated by a near twofold increase in urine osmolality concentration on day 11^38^. This study also demonstrated that this increased urine osmolality in the older group was highly correlated with decreased cognitive processing time (*r* = 0.79; *P* < 0.05). From such studies, it is hypothesized that as individuals age, maintaining serum osmolarity becomes more challenging, leading to high osmolarity, which in turn may be linked to cognitive function. However, it’s worth noting that most of these studies have primarily examined situations of moderate and tentative dehydration induced through activities like exercise, and there is limited research on the effects of repeated or sustained elevation in serum osmolarity on cognitive outcomes. In contrast to transient osmotic changes causing temporary cognitive effects, our current study demonstrated that repeated elevation of serum osmotic pressure across two time points was cross-sectionally associated with MoCA-J ≤ 22, suggesting a possible role for distinct physiological mechanisms in cognitive aging.

Usually, serum osmolarity tends to increase with aging^35^. As a potential cause, aging leads to a decrease in total body water volume^39^, making it more challenging to maintain a balance between fluid intake and excretion, resulting in a propensity for high osmolarity^40^. For instance, a study reported a dehydration prevalence of 24% in individuals aged 65 and older^33^, and another study found a prevalence of 36.4% in vulnerable community-dwelling older people in Japan (with an average age of 84 years)^16^. However, no individuals reported symptoms related to dehydration in these studies including the present study, in which the proportion of individuals (mean age 71 years) with serum osmotic pressure above 300 mOsm/L was 28%. Therefore, it is presumed that serum osmolarity increases with age without the individual’s awareness, potentially resulting in sustained elevation of serum osmolarity over time. This asymptomatic elevation may go unnoticed in routine clinical settings, underscoring the importance of monitoring serum osmolarity in older populations.

Based on the current study and others, it is reasonable to hypothesize that sustained elevation of serum osmolarity may be associated with cognitive decline. However, there are no studies that directly clarify the mechanisms by which elevated serum osmolarity might influence cognitive function. The involvement of osmotic stress is considered one plausible mechanism. An increase in serum osmotic pressure is considered to place various types of stress on cells. When the extracellular environment becomes hypertonic due to high serum osmotic pressure, water within the cell moves out, leading to an increase in intracellular solute concentration and a decrease in cell membrane tension, leading to cell shrinkage. In response, channels and other membrane proteins that sense membrane tension become activated^41^. These changes can disrupt higher-order protein functions within the cell and impair enzymatic reactions^41^. Furthermore, hypertonic stress triggers a stress response, activating the mitogen-activated protein kinase (MAPK) pathway^42^. The cellular responses resulting from this osmotic stress include short-term regulation of ion channels and transporters aimed at restoring cell volume due to water influx or efflux caused by osmotic changes^41^. Over the long term, cells adapt to sustained hypertonic external environments (Δ10–30 mOsm/kg for 48 h) through altered gene expression mediated by transcription factors, particularly nuclear factor of activated T-cells 5 (NFAT5), also known as tonicity-responsive enhancer binding protein (TonEBP), enabling them to survive even under osmotically stressful conditions^43–45^. On the other hand, if adaptation is not possible, it leads to apoptosis^46^. These findings indicate that both the effects of hyperosmolarity and adaptation to it involve profound alterations of the state of the cells^43^. In addition, as cells age, they accumulate stress from external factors such as heat, oxidative stress, and osmotic stress. This kind of stress can lead to the disruption of proteostasis^47^. For instance, while mitochondria are transported along axons, a reduction in axonal flow has been shown to lead to the disappearance or shrinkage of mitochondria within the axon^48^. The reduction of mitochondria within axons disrupts proteostasis, leading to decreased autophagy. Consequently, abnormal protein accumulation also occurs^49^. Given that these reactions become more pronounced with sustained osmotic stress, elevated serum osmolarity may play a role in ‘age-related cognitive decline’ and could be relevant to age-dependent pathological cognitive impairments such as Alzheimer’s disease.

To prevent sustained elevation of serum osmotic pressure, it is considered important to manage serum osmotic pressure on a daily basis. A prompt and effective method to prevent high osmotic pressure is to increase water intake; however, recognizing high serum osmotic pressure, especially for the elderly, is challenging^9,16,50^. In other words, it is difficult to compensate for persistently elevated serum osmotic pressure through subjective symptoms, and consequently, ongoing osmotic stress on neurons may persist without awareness. As a measure to avoid this, consciously increasing water intake rather than relying on subjective symptoms can be considered. Nevertheless, in the present study, no correlation was observed between daily NAD intake and osmotic pressure, nor was there any relationship with lower cognitive performance. These results may have been influenced by fasting from the night before or the composition of NAD. Furthermore, factors beyond NAD intake, including exercise and associated sweating, diet, environmental temperature, and humidity as well as the underlying health condition of each individual, may also have contributed to the serum osmotic pressure even in these relatively healthy individuals. Further research is required to establish effective methods for maintaining serum osmotic pressure at appropriate levels.

This study has several limitations. First, MoCA-J cognitive screening was conducted only between 2016 and 2020, preventing direct assessment of changes in cognitive performance over time. Without serial measures, we cannot establish intra-individual trajectories or distinguish between preexisting impairment and subsequent decline. This constraint particularly affects interpretation of associations with serum osmolarity measured in the 2012–2013 survey, as cognitive status at that time remains unknown. Second, serum osmotic pressure was estimated as calculated osmolarity in the 2012–2013 survey and measured directly as osmolality in the 2016–2017 survey, using different thresholds to define “high” values. These measurement differences limit direct intra-individual comparisons of osmotic pressure across time. Third, daily NAD intake was self-reported and used as a proxy for hydration behavior. Such self-administered questionnaires may not accurately capture total fluid intake, thirst perception, or underlying physiological hydration status. Fourth, although we adjusted for a range of demographic, lifestyle, and clinical covariates, residual confounding cannot be excluded. Unmeasured factors—such as dietary composition, physical activity level, ambient environmental conditions (temperature, humidity), renal function, and subclinical illnesses—may have influenced serum osmotic pressure and cognitive outcomes. Finally, this study was constrained by the limited variables available in the existing dataset, which restricted the scope of our analyses. Specifically, the small number of participants in the high-osmolarity group reduced our statistical power to detect modest effect sizes and resulted in wider confidence intervals for some estimates. As a result, the potential for Type II error remains, and caution is advised when generalizing these findings to other populations or clinical settings with different characteristics. Future validation in larger cohorts with more detailed phenotypic data is warranted.

In conclusion, this study revealed that repeated elevation of serum osmotic pressure across two time points was cross-sectionally associated with lower cognitive performance in the general elderly population, whereas no significant association was observed between daily NAD intake and serum osmotic pressure or cognitive function. Although daily fluid intake was not significantly associated with serum osmolarity or cognitive performance in this study, adequate hydration remains important for preventing dehydration, which is known to negatively affect physiological and cognitive functions. Given the absence of significant associations in this study, we avoided making specific claims regarding hydration or dietary factors. Further research is warranted to clarify the mechanisms underlying serum osmolarity and its potential impact on cognitive aging. Longitudinal studies are needed to confirm causality and validate these findings in broader populations.

## Supplementary Information

Below is the link to the electronic supplementary material.

Supplementary Material 1

## Data Availability

The most relevant data are within the paper. Raw data cannot be made publicly available, as study participants did not consent to have their information freely accessible. Based on these consents, the Ethics Committee of the Institute of Biomedical Research and Innovation at the Kobe Biomedical Innovation Cluster (approval no. 10–20) and Keio University School of Medicine inhibit any public data sharing because data contain potentially identifying or sensitive disease information. Data accession requests may be sent to the administration of the Ethics Committees. The data will be shared after a review of the purpose and with permission from the ethics committees. Data requests can be made to the corresponding author.

## References

[1] Livingston, G. et al. Dementia prevention, intervention, and care: 2020 report of the lancet commission. *Lancet* **396**, 413–446 (2020). 32738937 10.1016/S0140-6736(20)30367-6 PMC7392084

[2] Zhu, J. et al. Physical and mental Activity, disease Susceptibility, and risk of dementia: A prospective cohort study based on UK biobank. *Neurology* **99**, e799–e813 (2022). 35896434 10.1212/WNL.0000000000200701 PMC9484730

[3] Scarmeas, N., Anastasiou, C. A. & Yannakoulia, M. Nutrition and prevention of cognitive impairment. *Lancet Neurol.* **17**, 1006–1015 (2018). 30244829 10.1016/S1474-4422(18)30338-7

[4] Vandentorren, S. et al. Mortality in 13 French cities during the August 2003 heat wave. *Am. J. Public. Health*. **94**, 1518–1520 (2004). 15333306 10.2105/ajph.94.9.1518 PMC1448485

[5] Hajat, S., O’Connor, M. & Kosatsky, T. Health effects of hot weather: from awareness of risk factors to effective health protection. *Lancet* **375**, 856–863 (2010). 20153519 10.1016/S0140-6736(09)61711-6

[6] Millyard, A., Layden, J. D., Pyne, D. B., Edwards, A. M. & Bloxham, S. R. Impairments to thermoregulation in the elderly during heat exposure events. *Gerontol. Geriatr. Med.* **6**, 2333721420932432 (2020). 32596421 10.1177/2333721420932432 PMC7297481

[7] Cheuvront, S. N. & Kenefick, R. W. Dehydration: physiology, assessment, and performance effects. *Compr. Physiol.* **4**, 257–285 (2014). 24692140 10.1002/cphy.c130017

[8] Hooper, L., Bunn, D., Jimoh, F. O. & Fairweather-Tait, S. J. Water-loss dehydration and aging. *Mech. Ageing Dev.* **136–137**, 50–58 (2014). 24333321 10.1016/j.mad.2013.11.009

[9] Lambert, K. & Carey, S. Dehydration in geriatrics: consequences and practical guidelines. *Curr. Opin. Clin. Nutr. Metab. Care*. **26**, 36–41 (2023). 36131635 10.1097/MCO.0000000000000880

[10] Jequier, E. & Constant, F. Water as an essential nutrient: the physiological basis of hydration. *Eur. J. Clin. Nutr.* **64**, 115–123 (2010). 19724292 10.1038/ejcn.2009.111

[11] D’Anci, K. E., Constant, F. & Rosenberg, I. H. Hydration and cognitive function in children. *Nutr. Rev.* **64**, 457–464 (2006). 17063927 10.1301/nr.2006.oct.457-464

[12] Masento, N. A., Golightly, M., Field, D. T., Butler, L. T. & van Reekum, C. M. Effects of hydration status on cognitive performance and mood. *Br. J. Nutr.* **111**, 1841–1852 (2014). 24480458 10.1017/S0007114513004455

[13] Suhr, J. A., Hall, J., Patterson, S. M. & Niinisto, R. T. The relation of hydration status to cognitive performance in healthy older adults. *Int. J. Psychophysiology: Official J. Int. Organ. Psychophysiol.* **53**, 121–125 (2004). 10.1016/j.ijpsycho.2004.03.003 15210289

[14] Bethancourt, H. J., Kenney, W. L., Almeida, D. M. & Rosinger, A. Y. Cognitive performance in relation to hydration status and water intake among older adults, NHANES 2011–2014. *Eur. J. Nutr.* **59**, 3133–3148 (2020). 31776660 10.1007/s00394-019-02152-9 PMC8841102

[15] Nishi, S. K. et al. Water intake, hydration status and 2-year changes in cognitive performance: a prospective cohort study. *BMC Med.* **21**, 82 (2023). 36882739 10.1186/s12916-023-02771-4 PMC9993798

[16] Higashimura, S., Tamai, N., Nakagami, G., Tobe, H. & Sanada, H. A pilot epidemiological study on chronic dehydration of older adults in home care setting. *J. Nurs. Sci. Eng.* **9**, 123–135 (2022).

[17] Tanaka, S. et al. Impact of female sex on the susceptibility to hypernatremia among older Community-Dwelling individuals in Japan. *Int. J. Gen. Med.* **15**, 777–785 (2022). 35082525 10.2147/IJGM.S345150 PMC8786365

[18] Bialecka-Debek, A. & Pietruszka, B. The association between hydration status and cognitive function among free-living elderly volunteers. *Aging Clin. Exp. Res.* **31**, 695–703 (2019). 30128663 10.1007/s40520-018-1019-5 PMC6491399

[19] Takakura, Y., Otsuki, M., Takagi, R. & Houkin, K. A validation study for wide-range remote assessment of cognitive functions in the healthy older Japanese population: a pilot randomised crossover trial. *BMC Geriatr.* **23**, 575 (2023). 37723429 10.1186/s12877-023-04275-5 PMC10507887

[20] Nakagoshi, N. et al. Correction to: Determinants of double product: a cross-sectional study of urban residents in Japan. *Environ. Health Prev. Med.* **28**, 74 (2023). 38057093 10.1265/ehpm.23-00220 PMC10711371

[21] Higashiyama, A. et al. Does high-sensitivity C-reactive protein or low-density lipoprotein cholesterol show a stronger relationship with The cardio-ankle vascular index in healthy community dwellers? The KOBE study. *J. Atheroscler. Thromb.* **19**, 1027–1034 (2012). 22785137 10.5551/jat.13599

[22] Hirata, T. et al. HOMA-IR values are associated with glycemic control in Japanese subjects without diabetes or obesity: the KOBE study. *J. Epidemiol/Jpn. Epidemiol. Assoc.* **25**, 407–414 (2015). 10.2188/jea.JE20140172 PMC4444494 26005064

[23] Sugiyama, D. et al. The relationship between Lectin-Like oxidized Low-Density lipoprotein Receptor-1 ligands containing Apolipoprotein B and the Cardio-Ankle vascular index in healthy community inhabitants: the KOBE study. *J. Atheroscler. Thromb.* **22**, 499–508 (2015). 25374294 10.5551/jat.26450

[24] Kubota, Y. et al. Serum polyunsaturated fatty acid composition and serum High-Sensitivity C-Reactive protein levels in healthy Japanese residents: the KOBE study. *J. Nutr. Health Aging*. **19**, 719–728 (2015).26193854 10.1007/s12603-015-0497-9

[25] Tatsumi, Y. et al. Underweight young women without later weight gain are at high risk for osteopenia after midlife: the KOBE study. *J. Epidemiol/Jpn. Epidemiol. Assoc.* **26**, 572–578 (2016). 10.2188/jea.JE20150267 PMC5083320 27108753

[26] Agency, J. M. & Weather Japan Meteorological Agency:https://www.jma.go.jp/jma/indexe.html

[27] Nishikawa, T. et al. Daily habit of water intake in patients with cerebral infarction before its Onset; comparison with a healthy population: A Cross-Sectional study. *Cerebrovasc. Dis.* :1–8. (2019). 10.1159/000500075 31055576

[28] Worthley, L. I., Guerin, M. & Pain, R. W. For calculating osmolality, the simplest formula is the best. *Anaesth. Intensive Care*. **15**, 199–202 (1987). 3605570 10.1177/0310057X8701500214

[29] Hooper, L. et al. Diagnostic accuracy of calculated serum osmolarity to predict dehydration in older people: adding value to pathology laboratory reports. *BMJ Open.* **5**, e008846 (2015). 26490100 10.1136/bmjopen-2015-008846 PMC4636668

[30] Rasouli, M. Basic concepts and practical equations on osmolality: biochemical approach. *Clin. Biochem.* **49**, 936–941 (2016). 27343561 10.1016/j.clinbiochem.2016.06.001

[31] Ng, T. P. et al. Montreal cognitive assessment for screening mild cognitive impairment: variations in test performance and scores by education in Singapore. *Dement. Geriatr. Cogn. Disord*. **39**, 176–185 (2015). 25572449 10.1159/000368827

[32] Nara, M. et al. Japanese version of the Montreal cognitive assessment cut-off score to clarify improvement of mild cognitive impairment after exercise training in community-dwelling older adults. *Geriatr. Gerontol. Int.* **18**, 833–838 (2018). 29392877 10.1111/ggi.13253

[33] Parkinson, E. et al. Low-intake dehydration prevalence in non-hospitalised older adults: systematic review and meta-analysis. *Clin. Nutr.* **42**, 1510–1520 (2023). 37330324 10.1016/j.clnu.2023.06.010

[34] Okamura, T. et al. Japan atherosclerosis society (JAS) guidelines for prevention of atherosclerotic cardiovascular diseases 2022. *J. Atheroscler. Thromb.* **31**, 641–853 (2024). 38123343 10.5551/jat.GL2022 PMC11150976

[35] Nishikawa, T. et al. Seasonal variation in vascular dehydration risk: insights from the Kobe orthopedic and biomedical epidemiologic (KOBE) study. *Environ. Health Prev. Med.* **29**, 62 (2024). 39496441 10.1265/ehpm.24-00132 PMC11551438

[36] Pross, N. Effects of dehydration on brain functioning: A Life-Span perspective. *Ann. Nutr. Metab.* **70** (Suppl 1), 30–36 (2017). 28614811 10.1159/000463060

[37] Li, S., Xiao, X. & Zhang, X. Hydration status in older adults: current knowledge and future challenges. Nutrients 2023;15. 10.3390/nu15112609 PMC10255140 37299572

[38] Ainslie, P. N. et al. Energy balance, metabolism, hydration, and performance during strenuous hill walking: the effect of age. J Appl Physiol 2002;93:714–723. (1985). 10.1152/japplphysiol.01249.2001 12133883

[39] Luckey, A. E. & Parsa, C. J. Fluid and electrolytes in the aged. *Arch. Surg.* **138**, 1055–1060 (2003). 14557120 10.1001/archsurg.138.10.1055

[40] Lu, H., Ayers, E., Patel, P. & Mattoo, T. K. Body water percentage from childhood to old age. *Kidney Res. Clin. Pract.* **42**, 340–348 (2023). 37313612 10.23876/j.krcp.22.062 PMC10265208

[41] Hoffmann, E. K., Lambert, I. H. & Pedersen, S. F. Physiology of cell volume regulation in vertebrates. *Physiol. Rev.* **89**, 193–277 (2009). 19126758 10.1152/physrev.00037.2007

[42] Naguro, I. et al. ASK3 responds to osmotic stress and regulates blood pressure by suppressing WNK1-SPAK/OSR1 signaling in the kidney. *Nat. Commun.* **3**, 1285 (2012). 23250415 10.1038/ncomms2283

[43] Burg, M. B., Ferraris, J. D. & Dmitrieva, N. I. Cellular response to hyperosmotic stresses. *Physiol. Rev.* **87**, 1441–1474 (2007). 17928589 10.1152/physrev.00056.2006

[44] Brewster, J. L. & Gustin, M. C. Hog1: 20 years of discovery and impact. *Sci. Signal.* **7**, re7 (2014). 25227612 10.1126/scisignal.2005458

[45] Sumida, T. S. Hyperosmotic stress response regulates interstitial homeostasis and pathogenic inflammation. *J. Biochem.* **173**, 159–166 (2023). 36722164 10.1093/jb/mvad009

[46] Brocker, C., Thompson, D. C. & Vasiliou, V. The role of hyperosmotic stress in inflammation and disease. *Biomol. Concepts*. **3**, 345–364 (2012). 22977648 10.1515/bmc-2012-0001 PMC3438915

[47] Lopez-Otin, C., Blasco, M. A., Partridge, L., Serrano, M. & Kroemer, G. The hallmarks of aging. *Cell* **153**, 1194–1217 (2013). 23746838 10.1016/j.cell.2013.05.039 PMC3836174

[48] Takihara, Y. et al. In vivo imaging of axonal transport of mitochondria in the diseased and aged mammalian CNS. *Proc. Natl. Acad. Sci. U S A*. **112**, 10515–10520 (2015). 26240337 10.1073/pnas.1509879112 PMC4547257

[49] Shinno, K., Miura, Y., Iijima, K. M., Suzuki, E. & Ando, K. Axonal distribution of mitochondria maintains neuronal autophagy during aging via eIF2beta. bioRxiv. (2024). 10.7554/eLife.95576 PMC12834499 41587080

[50] Begg, D. P. Disturbances of thirst and fluid balance associated with aging. *Physiol. Behav.* **178**, 28–34 (2017). 28267585 10.1016/j.physbeh.2017.03.003

