# Supplementary material for: Association of repeated high serum osmolarity with cognitive function in older Japanese adults in a KOBE study subanalysis
Source: Sci Rep. 2025 Oct 21;15:36784. doi: 10.1038/s41598-025-20607-4 (PMC12540944; doi:10.1038/s41598-025-20607-4)
Supplement: Supplementary file 1 — Supplementary Material 1 [file 41598_2025_20607_MOESM1_ESM.docx]

| Supplementary Table S1. Participant characteristics stratified by serum osmolarity categories across surveys | | | | | | | | | | | | | | | | | | | | | | |
| --- | --- | --- | --- | --- | --- | --- | --- | --- | --- | --- | --- | --- | --- | --- | --- | --- | --- | --- | --- | --- | --- | --- |
|  |  |  |  | S.D. | Range | | |  |  |  | S.D. | Range | | |  |  |  | S.D. | Range | | | p value |
| The MoCA-J in the surveys (2016-2020) | Number | 154 (72.0%) | | |  | | |  | 60 (28.0%) | | |  | | |  | 214 | | |  | | |  |
|  | MoCA-J | 24.8 | ± | 3.1 | 14.0 | - | 30.0 |  | 23.8 | ± | 3.6 | 13.0 | - | 30.0 |  | 24.5 | ± | 3.2 | 13.0 | - | 30.0 |  |
|  | Age at MoCA-J | 76.1 | ± | 1.3 | 75 | - | 80 |  | 76.6 | ± | 1.4 | 75 | - | 80 |  | 76.3 | ± | 1.3 | 75 | - | 80 |  |
| The 2012-2013 survey | Serum Osmolarity | <300mOsm/L | | |  |  |  |  | >=300mOsm/L | | |  |  |  |  | Total |  |  |  |  |  |  |
|  | Age | 71.2 | ± | 2.0 | 67 | - | 76 |  | 71.7 | ± | 2.3 | 67 | - | 76 |  | 71.3 | ± | 2.1 | 67 | - | 76 | n.s. |
|  | Female sex (%) | 83 (53.9%) | | |  |  |  |  | 37 (61.8%) | | |  |  |  |  |  |  |  | 120 (56.1%) | | | n.s. |
|  | Height | 158.7 | ± | 7.5 | 142.9 | - | 176.7 |  | 156.9 | ± | 8.7 | 140.9 | - | 177.1 |  | 158.2 | ± | 7.8 | 140.9 | - | 177.1 | n.s. |
|  | Weight | 55.9 | ± | 9.4 | 35.9 | - | 83.2 |  | 52.9 | ± | 9.6 | 37.9 | - | 82.1 |  | 55.1 | ± | 9.5 | 35.9 | - | 83.2 | 0.038 |
|  | Smoker at the survey | 4 (2.6%) | | |  |  |  |  | 2 (3.3%) | | |  |  |  |  | 6 (2.8%) | | |  |  |  | n.s. |
|  | Drinker at the survey | 82 (53.2%) | | |  |  |  |  | 24 (40.0%) | | |  |  |  |  | 106 (49.5%) | | |  |  |  | n.s. |
|  | SBP (mmHg) | 119.7 | ± | 17.6 | 75.0 | - | 174.0 |  | 120.2 | ± | 16.9 | 90.0 | - | 161.0 |  | 119.9 | ± | 17.4 | 75.0 | - | 174.0 | n.s. |
|  | DBP (mmHg) | 71.9 | ± | 10.3 | 45.0 | - | 106.0 |  | 72.9 | ± | 11.0 | 48.0 | - | 102.0 |  | 72.2 | ± | 10.5 | 45.0 | - | 106.0 | n.s. |
|  | Hypertension | 37 (24.0%) | | |  |  |  |  | 12 (20.0%) | | |  |  |  |  | 49 (22.9%) | | |  |  |  | n.s. |
|  | Diabetes | 6 (3.9%) | | |  |  |  |  | 2 (3.3%) | | |  |  |  |  | 8 (3.7%) | | |  |  |  | n.s. |
|  | Dyslipidemia | 69 (44.8%) | | |  |  |  |  | 27 (45.0%) | | |  |  |  |  | 96 (44.9%) | | |  |  |  | n.s. |
|  | NAD intake (mL/day) | 1802.1 | ± | 700.1 | 450.0 | - | 4680.0 |  | 1688.8 | ± | 715.7 | 400.0 | - | 3550.0 |  | 1770.3 | ± | 704.6 | 400.0 | - | 4680.0 | n.s. |
| The 2016-2017 survey | Serum Osmolarity | <295mOsm/Kg | | |  |  |  |  | >=295mOsm/Kg | | |  |  |  |  | Total |  |  |  |  |  |  |
|  | Age | 75 | ± | 2 | 71 | - | 80 |  | 76 | ± | 2 | 72 | - | 79 |  | 75 | ± | 2 | 71 | - | 80 | n.s. |
|  | Female sex (%) | 110 (56.4%) | | |  |  |  |  | 3 (30.0%) | | |  |  |  |  | 113 (55.1%) | | |  |  |  | n.s. |
|  | Height | 157.3 | ± | 7.8 | 141.1 | - | 176.7 |  | 162.3 | ± | 9.9 | 145.9 | - | 176.0 |  | 157.4 | ± | 8.0 | 140.4 | - | 176.7 | n.s. |
|  | Weight | 54.5 | ± | 9.5 | 36.2 | - | 83.5 |  | 63.1 | ± | 13.5 | 47.4 | - | 82.6 |  | 55.0 | ± | 9.9 | 36.2 | - | 83.5 | 0.007 |
|  | Smoker at the survey | 5 (2.6%) | | |  |  |  |  | 1 (10.0%) | | |  |  |  |  | 6 (2.90%) | | |  |  |  | n.s. |
|  | Drinker at the survey | 98 (50.3%) | | |  |  |  |  | 5 (50.0%) | | |  |  |  |  | 103 (50.2%) | | |  |  |  | n.s. |
|  | SBP (mmHg) | 123.0 | ± | 18.4 | 81.0 | - | 171.0 |  | 120.9 | ± | 17.4 | 98.0 | - | 152.0 |  | 122.9 | ± | 18.4 | 81.0 | - | 171.0 | n.s. |
|  | DBP (mmHg) | 71.5 | ± | 10.1 | 47.0 | - | 99.0 |  | 69.9 | ± | 10.1 | 57.0 | - | 88.0 |  | 71.4 | ± | 10.1 | 47.0 | - | 99.0 | n.s. |
|  | Hypertension | 33 (16.9%) | | |  |  |  |  | 1 (10.0%) | | |  |  |  |  | 34 (16.6%) | | |  |  |  | n.s. |
|  | Diabetes | 12 (6.2%) | | |  |  |  |  | 0 (0.0%) | | |  |  |  |  | 12 (5.9%) | | |  |  |  | n.s. |
|  | Dyslipidemia | 92 (47.2%) | | |  |  |  |  | 7 (70.0%) | | |  |  |  |  | 99 (48.3%) | | |  |  |  | n.s. |
|  | NAD intake (mL/day) | 1683.5 | ± | 679.4 | 0.0 | - | 4350.0 |  | 1903.0 | ± | 690.7 | 1000.0 | - | 2750.0 |  | 1694.2 | ± | 679.9 | 0.0 | - | 4350.0 | n.s. |
| Continuous data was analyzed using student's t test and is shown in the mean ± standard deviation. S.D.; standard deviation. Categorical data was analyzed using the chi-square test and is shown as number (%). Hypertension; systolic blood pressure>=140mmHg, diastolic blood pressure>=90, or taking antihypertensives. Diabetes; fasting glucose >126 mg/dL, HbA1C >=6.5%, taking anti-diabetics medication or its history in the medical record. Dyslipidemia; LDL>=140mg/dL, triglyceride >=150mg/dL or HDL <40mg/dL. LDL; low density lipoprotein. HDL; high density lipoprotein. NAD; non-alcohol drink. MoCA-J; Japanese version of Montreal Cognitive Assessment, coducted between 2016 and 2020. n.s.: not significant | | | | | | | | | | | | | | | | | | | | | | |
